# *BMC Family Practice* reviewer acknowledgment 2015

**DOI:** 10.1186/s12875-016-0419-x

**Published:** 2016-02-12

**Authors:** Elaine Zhang

**Affiliations:** BioMed Central, Floor 6, 236 Gray’s Inn Road, London, WC1X 8HB UK

## Abstract

The editors of BMC Family Practice would like to thank all our reviewers who have contributed to the journal in Volume 16 (2015)

**Arye Abeles**

USA

**Oladele Vincent Adeniyi**

South Africa

**Girdhar Agarwal**

India

**Sarah Alderson**

UK

**Kelli Allen**

USA

**Muhammad Alotaibi**

Kuwait

**Grove Amy**

UK

**Foteini Anastasiou**

Greece

**Angeliki Angelidi**

Greece

**Aniela Angelow**

Germany

**Eva Arvidsson**

Sweden

**Diane Ashiru-Oredope**

UK

**Felicity Astin**

UK

**Salla Atkins**

Sweden

**Kris Aubrey-Bassler**

Canada

**Luciana Ballini**

Italy

**Paola Ballotari**

Italy

**Michelle Banfield**

Australia

**Yael Bar Zeev**

Israel

**Marie Barais**

France

**Elizabeth Barley**

UK

**Karen Barnett**

UK

**Heather Barry**

UK

**Christopher Barton**

Australia

**Maria Basta**

Greece

**Per Bech**

Denmark

**Iain Beith**

UK

**Ann Berger**

USA

**Christophe Berkhout**

France

**Annette Bishop**

UK

**Lindsay Blank**

UK

**Jutta Bleidorn**

Germany

**Jeanet Blom**

Netherlands

**Eva Blozik**

Germany

**Femke Boehmer**

Germany

**Wienke Boerma**

Netherlands

**Fiona Boland**

Ireland

**Gunnar Bondevik**

Norway

**S Boodai**

UK

**Helen P. Booth**

UK

**Marije Bosch**

Australia

**Marie Bradley**

USA

**Kate Brain**

UK

**Paula Brauer**

Canada

**Iain Brew**

UK

**Bianca Brijnath**

Australia

**Eileen Britt**

New Zealand

**B.D. Lidewij Broekhuizen**

Netherlands

**Lisa Brunton**

UK

**Alessandra Buja**

Italy

**Christopher Burton**

UK

**Joan Busfield**

UK

**Jo Byrne**

UK

**Paul Campbell**

UK

**Stephen Campbell**

UK

**Christine Carius**

Germany

**Melinda Carrington**

Australia

**Yasemin Çayır**

Turkey

**Venija Cerovecki**

Croatia

**Gülsen Ceyhun Peker**

Turkey

**Tom Chan**

UK

**Anna Cheshire**

UK

**Rie Chiba**

Japan

**Kyoung Im Cho**

Korea, South

**Fatma Cihan**

Turkey

**David J Clarke**

UK

**John Cleland**

UK

**Manuel J. Cobo**

Spain

**Naama Constantini**

USA

**Carol Coole**

UK

**Vincent Cooper**

UK

**Suzanne Cosh**

Australia

**Elizabeth Cottrell**

UK

**Peter Alan Coventry**

UK

**Alan Crockett**

Australia

**Jesse Crosson**

USA

**Sarah Cutrona**

USA

**Slawomir Czachowski**

Poland

**Carl D'Arcy**

Canada

**Eefje De Bont**

Netherlands

**Daan De Coster**

UK

**Corina De Jong**

Netherlands

**Jip De Jong**

Netherlands

**Jan De Maeseneer**

Belgium

**An De Sutter**

Belgium

**Marcelo Demarzo**

Brazil

**Catherine Demers**

Canada

**Sophie Derchain**

Brazil

**Tobias Deutsch**

Germany

**Fiona Doolan-Noble**

New Zealand

**Athanassios Douzenis**

Greece

**Frank Doyle**

Ireland

**Alex Dregan**

UK

**Sinead Duane**

Ireland

**Anthony D'Urzo**

Canada

**Anne G. Ekeland**

Norway

**Nouhad El-Haddad**

Australia

**Rose Ellis**

Australia

**Jon Emery**

Australia

**Romain Eschalier**

France

**Evangelos Evangelou**

Greece

**Karen Fairhurst**

UK

**Andrew Farmer**

UK

**Nighat Faruqi**

Australia

**Annunziata Faustini**

Italy

**Ahmad Fayaz-Bakhsh**

Iran

**Gregor Feldmeier**

Germany

**Julie Ferguson**

UK

**Bart Ferket**

USA

**Rachael Finn**

UK

**Emily Fletcher**

UK

**Michelle Foley**

UK

**Lynne Forrest**

UK

**Yvonne Forsell**

Sweden

**Nick Francis**

UK

**Toby Freeman**

Australia

**Thomas Frese**

Germany

**John Furler**

Australia

**Daniel Fuster**

Spain

**Ildiko Gagyor**

Germany

**Maria Garcia-Gil**

Spain

**Poonam Gardner-Sood**

UK

**Natalie Gauld**

New Zealand

**Chris Gibbons**

UK

**Christopher Gidlow**

UK

**Antonio Z Gimeno García**

Spain

**Carlo Bruno Giorda**

Italy

**Paolo Giorgi Rossi**

Italy

**Dianne Goeman**

Australia

**Katja Goetz**

Germany

**Fatma Gökşin Cihan**

Turkey

**Debora Goldberg**

USA

**Trish Gorely**

UK

**Martine Granek-Catarivas**

Israel

**Aileen Grant**

UK

**Marcia Grant**

USA

**Ben Gray**

New Zealand

**Eva Grunfeld**

Canada

**Anna Gryko**

Poland

**Jane Gunn**

Australia

**Aimin Guo**

China

**Anders Halling**

Denmark

**Janet Hanley**

UK

**Shamil Haroon**

UK

**Mark Harris**

Australia

**Jo Hart**

UK

**Johannes Hauswaldt**

Germany

**Rebecca Hays**

UK

**Christian Helfrich**

USA

**Erik Hemmingsson**

Sweden

**Wolfgang Himmel**

Germany

**Samuel Ho**

USA

**Robert Hoffman**

Israel

**Rhona Hogg**

UK

**Knut Holtedahl**

Norway

**Guro Huby**

Norway

**John Hughes**

UK

**Kate Hunt**

UK

**Deirdre Hurley Osing**

Ireland

**Sylvia Hysong**

USA

**Eliza Iatraki**

Greece

**Fumiaki Imamura**

UK

**Baruch Itzhak**

Israel

**Pupalan Iyngkaran**

Australia

**Sally Jacobs**

UK

**Masahito Jimbo**

USA

**Hanna Kaduszkiewicz**

Germany

**Vera Kalitzkus**

Germany

**Sebastian Kalwij**

UK

**Khaled Karkabi**

Israel

**Marise Kasteleyn**

Netherlands

**Paul Kelly**

UK

**Jakub Kenig**

Poland

**Anne Kennedy**

UK

**Natalie Kennie-Kaulbach**

Canada

**Cassandra Kenning**

UK

**Amanda Kenny**

Australia

**Myrna Keurhorst**

Netherlands

**Chris Keyworth**

UK

**Gholam Reza Kheirabadi**

Iran, Islamic Republic Of

**Moira Kinnear**

UK

**Bruce Kirenga**

Uganda

**Spyridon Klinis**

Greece

**Louise Knight**

UK

**Sarah Knowles**

UK

**Lee Yew Kong**

Malaysia

**Kurt Kroenke**

USA

**Edward Krupat**

USA

**Donata Kurpas**

Poland

**Uwe Kurzke**

Germany

**Cindy Lam**

Hong Kong

**Maaike Langelaan**

Netherlands

**Susanne Langer**

UK

**Birgitta Langhammer**

Norway

**Michel Langlois**

Belgium

**Miranda Laurant**

Netherlands

**Rachel Laws**

Australia

**Patricia Lebensohn**

USA

**Marieke B Lemiengre**

Belgium

**Wei Shen Lim**

UK

**Alyson Littman**

USA

**Carl Lombard**

South Africa

**Daniel Lopez-Cevallos**

USA

**Fabiana Lorencatto**

UK

**Marta Losa Iglesias**

Spain

**Alexander Lustman**

Israel

**Richard Ma**

UK

**Annabelle Machin**

UK

**Parker Magin**

Australia

**Elizabeth Magnan**

USA

**Nicola Magnavita**

Italy

**Chris Maher**

Australia

**Helle Terkildsen Maindal**

Denmark

**Lilach Malatskey**

Israel

**Navjeet Mangat**

UK

**Adelais Markaki**

Greece

**Gabriella Marx**

Germany

**Edita Masanauskiene**

Lithuania

**Neal Maskrey**

UK

**Ioannis Mastoris**

USA

**Andre Matalon**

Israel

**Shin Matsuoka**

Japan

**Milena Maule**

Italy

**Mary Mccarron**

Ireland

**Serena Mccluskey**

UK

**Ron Mcdowell**

Ireland

**Sarah Mclachlan**

UK

**Kumara Mendis**

Sri Lanka

**Gabriele Messina**

Italy

**Ilias Migdalis**

Greece

**Wendy Miller**

USA

**Mirella Minkman**

Netherlands

**Matthew D. Mitchell**

USA

**Erin Moore**

USA

**Andrew Morden**

UK

**Frank Moriarty**

Ireland

**Rebecca Morris**

UK

**Melanie Morris**

UK

**Luke Mounce**

UK

**Bozena Mroczek**

Poland

**Jean Muris**

Netherlands

**Edel Murphy**

Ireland

**Lucio Naccarella**

Australia

**Cristiana Nascimento-Carvalho**

Brazil

**Pauline Nelson**

UK

**David Neubauer**

USA

**Eva Neufeld**

Canada

**Evangelia Ntzani**

Greece

**Peter Nugus**

Canada

**Austin O Carroll**

Ireland

**Brendan O Shea**

Ireland

**Maeve O'Beirne**

Canada

**Allison Ober**

USA

**Amy O'Donnell**

UK

**Nelly Oelke**

Canada

**Jodie Oliver-Baxter**

Australia

**Braden O'Neill**

Canada

**Juan Fco Orueta**

Spain

**Brendan O'Shea**

Ireland

**Alice Owen**

Australia

**Alis Özçakır**

Turkey

**Mehmet Özen**

Turkey

**Zlata Ozvacic Adzic**

Croatia

**Leonidas Palaiodimos**

Greece

**Maria Panagioti**

UK

**Kosmas Paraskevas**

Greece

**Deborah Parra-Medina**

USA

**Zoe Paskins**

UK

**Winifred Paulis**

Netherlands

**Christina Pearce**

UK

**Emily Peckham**

UK

**Marta Pelayo**

Spain

**Michael Pentzek**

Germany

**Giulio Perugi**

Italy

**Davorina Petek**

Slovenia

**Marija Petek ¿Ter**

Slovenia

**Ferdinando Petrazzuoli**

Sweden

**Ferdinando Petrazzuoli**

Italy

**Mila Petrova**

UK

**Carlo Piccinni**

Italy

**Tamar Pincus**

UK

**Hilary Pinnock**

UK

**Maria Carmen Portillo**

Spain

**Rebekah Pratt**

USA

**Ivanka Prichard**

Australia

**Emmanuel Prokopakis**

Greece

**Sue Pullon**

New Zealand

**Martine Puts**

Canada

**Jennifer Quint**

UK

**Pietro Ragni**

Italy

**Vasiliki Rahimzadeh**

Canada

**Anil Rane**

India

**Elena Ratschen**

UK

**Robin Ray**

Australia

**Genevieve Rayner**

Australia

**Sabi Redwood**

UK

**Carole Reeve**

Australia

**Joanne Reeve**

UK

**David Reeves**

UK

**Darlene Reid**

Canada

**Zeljko Reiner**

Croatia

**Cristina Renzi**

UK

**Suzanne Richards**

UK

**Eckhard Rickels**

Germany

**Herbert Riechelmann**

Austria

**Diane Roberts**

UK

**Charo Rodriguez**

Canada

**Anne Elizabeth Rogers**

UK

**Aldo Rosano**

Italy

**Marianne Rosendal**

Denmark

**Danica Rotar-Pavlic**

Slovenia

**Eugene Ruzagira**

Uganda

**Caroline Sanders**

UK

**Sally Sargeant**

Australia

**Kathleen Sarmiento**

USA

**Willemijn Schäfer**

Netherlands

**Jeffrey Scherrer**

USA

**Kathrin Schlößler**

Germany

**Guido Schmiemann**

Germany

**Antoinette Schoenthaler**

USA

**Antonis Schoinas**

Greece

**Jane Scott**

Australia

**Polona Selic**

Slovenia

**Turan Set**

Turkey

**Joshua Shadd**

Canada

**Michal Shani**

Israel

**James Sheppard**

UK

**Damin Si**

Australia

**Jonathan Silverman**

UK

**Rosemary Simmonds**

UK

**Panagiotis Simos**

Greece

**Aloysius Niroshan Siriwardena**

UK

**Susan Slade**

Australia

**Sarah Slight**

UK

**Nicola Small**

UK

**Susan Smith**

Ireland

**Sarah Smithson**

UK

**Emmanouil Smyrnakis**

Greece

**Ratna Sohanpal**

UK

**Robert Sokol**

USA

**Andreas Sonnichsen**

Germany

**Jaime Sousa**

Portugal

**Sheryl Spithoff**

Canada

**Melinda Stanners**

Australia

**Ellen Stein**

USA

**Aleksander Stepanović**

Slovenia

**Fiona Stevenson**

UK

**Scott Stewart**

USA

**Nigel Stocks**

Australia

**Matej Strnad**

Slovenia

**Andre Strydom**

UK

**Joachim Sturmberg**

Australia

**Frank Sullivan**

Canada

**Rachael Summers**

UK

**Patricia Sunaert**

Belgium

**Frederick Sundram**

New Zealand

**Elisabeth Svensson**

Sweden

**Emmanouil Symvoulakis**

Greece

**Adam G Tabak**

UK

**Mary R Talen**

USA

**Ryan Tandjung**

Switzerland

**Athina Tatsioni**

Greece

**Essa Tawfiq**

New Zealand

**Meredith Temple-Smith**

Australia

**Emma Thomas-Jones**

UK

**Hans Thulesius**

Sweden

**Petter Tinghog**

Sweden

**Els Tobback**

Belgium

**Gail Todd**

South Africa

**Pinar Topsever**

Turkey

**Helen Triantafyllidi**

Greece

**Katrina Tsang**

Hong Kong

**Hedda Tschudi-Madsen**

Norway

**Raymond Tweheyo**

UK

**Fiona Ulph**

UK

**Jonathan Underhill**

UK

**Christine Urquhart**

UK

**Michael Van Den Berg**

Netherlands

**Ann Van Den Bruel**

UK

**Pieter Van Den Hombergh**

Netherlands

**Johannes C Van Der Wouden**

Netherlands

**Nikki Van Dessel**

Netherlands

**Sonja Van Dillen**

Netherlands

**Sandra Van Dulmen**

Netherlands

**Guido Van Hal**

Belgium

**Oliver Van Hecke**

UK

**Harm Van Marwijk**

UK

**Paul Van Royen**

Belgium

**Tjeerd Van Staa**

UK

**Chris Van Weel**

Australia

**Isabelle Vedel**

Canada

**Jane Vennik**

UK

**Jan Verbakel**

Belgium

**Peter Verhaak**

Netherlands

**Theo Verheij**

Netherlands

**Areti Angeliki Veroniki**

Canada

**Evelyn Vingilis**

Canada

**Shlomo Vinker**

Israel

**Jaco Voorham**

Netherlands

**Adrian Wagg**

Canada

**Brian Walitt**

USA

**Anne Walsh**

Australia

**Harry Hao-Xiang Wang**

UK

**Shengyu Wang**

China

**Roger Webb**

UK

**Brandon Welch**

USA

**Matthias Wermeling**

Germany

**Alison While**

UK

**Anne Whittaker**

UK

**Sarah Wilke**

Netherlands

**Veronica Wilkie**

UK

**Emily Williams**

UK

**Adam Windak**

Poland

**Connie Wiskin**

UK

**Maria Wolters**

UK

**Carmen Wong**

Hong Kong

**Christopher Wong **

UK

**Rachael Wood**

UK

**Fiona Wood**

UK

**James Woodall**

UK

**Elizabeth Worsley**

UK

**Nat Wright**

UK

**Tina Wulff**

Germany

**Gwenllian Wynne-Jones**

UK

**Hakan Yaman**

Turkey

**John Yaphe**

Portugal

**Ferdi Yavuz**

Turkey

**Michael Yelland**

Australia

**Benjiamin Yip**

Hong Kong

**Zhaokang Yuan**

China

**Cristian Zeni**

USA

**Yinsheng Zhang**

China

**Andrzej Zielinski**

Sweden

**Nicholas Zwar**

Australia

